# Green Synthesis of Chitosan Silver Nanoparticle Composite Materials: A Comparative Study of Microwave and One-Pot Reduction Methods

**DOI:** 10.3390/polym17212960

**Published:** 2025-11-06

**Authors:** Ahmed Hosney, Algimanta Kundrotaitė, Donata Drapanauskaitė, Marius Urbonavičius, Šarūnas Varnagiris, Sana Ullah, Karolina Barčauskaitė

**Affiliations:** 1 Lithuanian Research Centre for Agriculture and Forestry, Instituto al. 1, Akademija, LT-58344 Kėdainiai District Municipality, Lithuania; algimanta.kundrotaite@lammc.lt (A.K.); donata.drapanauskaite@lammc.lt (D.D.); sana.ullah@lammc.lt (S.U.); 2 Center for Hydrogen Energy Technologies, Lithuanian Energy Institute, 3 Breslaujos, LT-44403 Kaunas, Lithuania

**Keywords:** chitosan, silver-nanoparticles, composite material, microwave-assisted, one-pot reduction

## Abstract

Green synthesis methods of silver nanoparticles have gained great attention because they offer sustainable, eco-friendly, and less-toxic alternatives to traditional methods. This study sheds light on the green synthesis of chitosan silver nanoparticle composites, providing a comparative evaluation of microwave-assisted (M1) and a one-pot (M2) reduction methods. The morphological, crystallinity, and structural uniformity characteristics were evaluated by UV-Visible, Raman spectroscopy, X-ray diffraction (XRD) and scanning electron microscopy (SEM) with employing image processing pipeline based on deep learning model for segmentation and particles size estimation. The UV-visible spectrum exhibited independent SPR peaks ranging from 400 to 450 nm for all samples; however, microwave assisted-synthesis possessed narrower and more intense peaks indicative of better crystallinity and mono-dispersity. SEM depicted smaller, more uniformly dispersed particles for microwave-assisted (M1), while deep learning segmentation showed lower particle size variability (σ ≈ 24–43 nm), compared to polydisperse (σ ≈ 16–59 nm) in M2 samples. XRD showed crystalline face-centered cubic (FCC) silver with dominant peaks in M1 samples, whereas M2 had broader, less intense peaks with amorphous features. Raman vibrations revealed more structural order and homogenous capping in M1 than M2. Therefore, microwave-assisted (M1) showed better control on nucleation, particle size, crystallinity, and homogeneity due to a faster and uniform energy distribution. The future research would focus on the antimicrobial evaluation of such nanoparticles in agronomy.

## 1. Introduction

The green synthesis and development of metal nanoparticles have gained significant attention due to their unique physical, chemical, and biological properties that distinguish them from their bulk counterparts [1,2,3,4,5,6,7]. Silver nanoparticles, in particular, have attracted considerable interest among various types of metal nanoparticles because of their ability to create innovative and advanced functional materials, reflecting their optical, catalytic, and antimicrobial properties [3,8,9]. Additionally, chitosan silver nanoparticles (Ch-AgNPs) have demonstrated impressive properties and hold potential for developing new drug delivery systems, antimicrobial agents, biomaterials, tissue regeneration, and coatings for medical devices [1,10,11,12,13]. Natural reducing agents, such as organic or plant extracts, are widely used in biosynthesis methods and are environmentally friendly compared to traditional chemical approaches. The lack of toxic by-products and the preference for biopolymers like chitosan enhance safety, making these nanoparticles suitable for biomedical applications [13]. Green synthesis methods for obtaining chitosan-silver nanoparticles (Ch-AgNPs) offer many advantages, mainly due to their environmental benefits and improved functional properties. These methods utilize natural materials and processes, avoiding harmful chemicals and energy-intensive procedures. Moreover, chitosan acts as a capping agent, providing additional biocompatibility and stability to the nanoparticles, which allows for safe applications across various fields [4,12,13,14,15,16,17].

Chitosan, a natural biopolymer produced from chitin in crustacean shells, insects, and fungi, has become a substantial component in the sustainable production of nanomaterials due to its biocompatibility, biodegradability, and non-toxicity [18,19,20]. Shrimp shells are the main global source of chitin and chitosan production [21,22,23,24,25,26]. Extraction of chitosan from shrimp shells is the most viable, sustainable, and eco-friendly option for treating these large quantities of bio-waste shells, as opposed to burning or dumping them in landfills, which attracts pathogenic microorganisms and alters soil properties [27]. Chitosan metal nanomaterials have much larger surface areas per volume unit than their raw bulk materials, indicating their size transition from bulk materials to nanomaterials, reflecting their change in the optical, mechanical, and dominance of surface characteristics, which in turn increases the chemical and biological reactivity of the material, making them ideal for diverse applications [13,28,29,30,31,32,33]. The large free hydroxyl and amino groups in chitosan possess the ability to reduce metal salts into metal nanoparticles [34,35,36,37], whereas its deacetylation degree (DD) plays a key role in the synthesis of chitosan metal nanomaterials and affects its antimicrobial properties [8,38,39]. When chitosan pairs with silver nanoparticles, which are known for their significant antimicrobial properties, the resulting product of chitosan-silver nanocomposites (Ch-AgNPs) unlocks synergistic effects that can tackle the pressing challenges in agriculture, food, environmental remediation, and the healthcare industry [4,12,31,32,40,41].

There are several developed methods for synthesizing nanoparticles using physical and chemical methods [4,31,33,37,42,43,44,45]. However, physical methods such as radiolysis, aerosol, and laser ablation techniques are efficient in silver nanoparticle preparation; they are costly and energy ineffective. In contrast, the chemical reduction of silver salts by organic solvents or extracts is the most widely applied approach for the synthesis of silver nanoparticles due to its eco-friendliness, cost-effectiveness, and applicability in large-scale production [17,35,36]. Silver nanoparticle synthesis has been developed using chitosan as both a reducing and capping agent, and this approach has proven to be more effective than the conventional method, which involves toxic chemical agents [16,17,29]. Previous studies reported that the reduction of silver salts can be achieved using chitosan and reducing agents such as ascorbic acid, hydrazine, sodium borohydride, and sodium citrate, paying attention to the toxicity and environmental limits. Ascorbic acid is the most frequently applied low-toxicity, reducing, and stabilizing agent in the synthesis of chitosan-silver nanoparticles [1,11,46].

Two of the most used green methods in the synthesis of chitosan-based metallic nanoparticles are microwave-assisted synthesis and one-pot reduction, valued for their energy efficiency, reaction kinetics, and overall nanoparticle characteristics [17,29,41]. The microwave-assisted method involves rapid and uniform volumetric heating, which is efficient and promotes faster nanoparticle growth, often resulting in improved particle properties [33,42,45]. Conversely, the one-pot reduction method is typically carried out at low temperatures using chemical reductants like sodium borohydride, producing nanoparticles with distinctive features. Chitosan plays a dual role in both approaches as a stabilizer and a reducing agent, enabling environmentally friendly synthesis with fewer hazardous reagents [1,17,36].

Despite the widespread use of both synthesis strategies in chitosan-based metallic nanoparticle production, there is a lack of direct comparisons of how each strategy affects nanoparticle characteristics using the same precursor. A comparative study is needed to guide method selection in agricultural, environmental, and biomedical fields, where the size, crystallinity, and stability of nanoparticles greatly impact their functional performance.

Therefore, the present research provides a comparative evaluation of chitosan-silver nanoparticle composite materials (Ch-AgNPs) synthesized via two distinct routes: microwave-assisted and one-pot reduction-based methods, and how the synthesis techniques dictate the nanoparticle properties within the chitosan matrix. In both routes, chitosan products derived from shrimp shells via different inorganic and organic demineralization and deproteinization optimization schemes were employed as a biopolymeric base for green synthesis. Accordingly, this study aims to assess the effect of synthesis techniques on the structural and physicochemical characteristics of the resultant Ch-AgNPs.

## 2. Materials and Methods

### 2.1. Materials

For this research, Hydrochloric acid (37%), lactic acid (96%), acetic acid (99%), and ascorbic acid (98%) were purchased from Merck (Darmstadt, Germany). Sodium borohydride (98%) from PanReac AppliChem (Monza, Italy), while sodium hydroxide (98%), and silver nitrate (≥99%) were purchased from VWR (Wayne, PA, USA).

### 2.2. Methods

**Figure 1:**
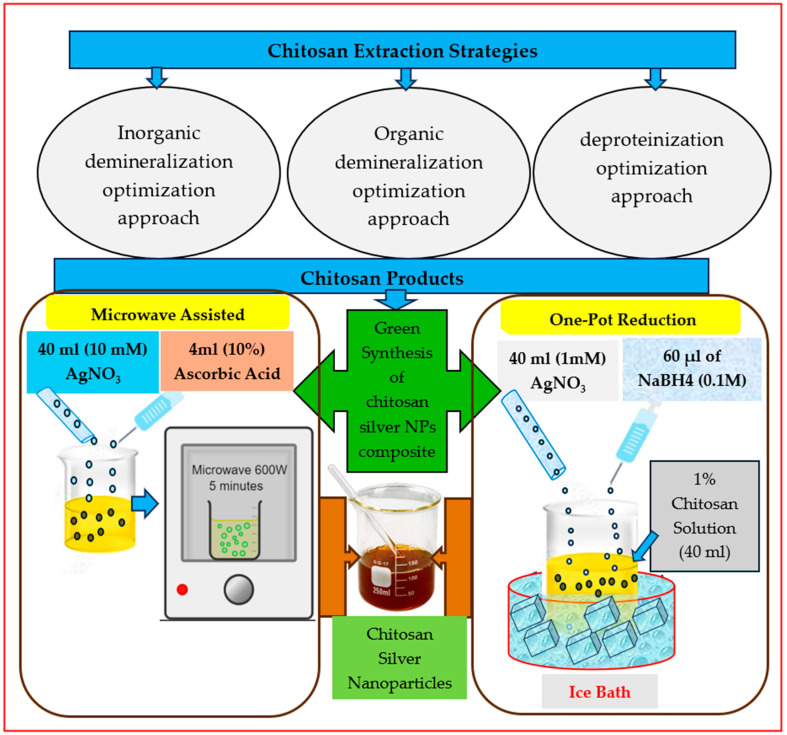
Graphical abstract of the biosynthesis of chitosan silver nanoparticle composite materials via microwave and one-pot reduction methods.

Chitosan-based silver nanoparticle composite materials were synthesized by the reduction of silver nitrate salt with chitosan recovered from shrimp shells using the following methodology, as represented in Figure 1.

#### 2.2.1. Preparation of Chitosan Solution

The characteristics of chitosan samples isolated from shrimp shells are depicted in Table 1. A 1% (*w*/*v*) chitosan solution was prepared by dissolving 2 g of chitosan powder in 2% (*v*/*v*) acetic acid. The same samples AS3, LH1, HC1, HC2, DP4, L10, and L20 of the prepared chitosan solutions were divided into two batches. The samples of the first batch were labeled in the following sequence: AS3.M1, LH1.M1, HC1.M1, HC2.M1, DP4.M1, L10.M1, L20.M1, and were used for nanoparticle synthesis by the microwave-assisted reduction method. Similarly, the samples of the second batch were named in sequence as AS3.M2, LH1.M2, HC1.M2, HC2.M2, DP4.M2, L10.M2, L20.M2, and used for nanoparticle synthesis by the one-pot reduction-based method.

#### 2.2.2. Chitosan-Silver Nanocomposite Synthesis

A. Method 1 (M1): Microwave-assisted reduction method

40 mL of 10 mM silver nitrate (AgNO_3_) solution was added to 40 mL of the prepared chitosan solution, then mixed with 4 mL of 10% (*w*/*v*) ascorbic acid, and then the PH was adjusted to 5.5. The reduction reaction was conducted under microwave irradiation at 600 watts for 5 min and then allowed to cool at room temperature. The collected Ch-Ag nanoparticles composites were centrifuged at 2000 rpm for 3 h, washed with distilled water, dried at 105 °C, and stored for further characterization analysis [47].

B. Method 2 (M2): One-pot reduction-based method

Chitosan silver nanoparticle composite materials were synthesized via a one-pot reduction of AgNO_3_ with chitosan (Ch), a model biopolymer, using NaBH4 as a stabilizer. The synthesis began by adding 1 mM AgNO_3_ to a 1% (*w*/*v*) Ch solution. The solution was stirred for 30 min while cooled in an ice bath. Afterwards, 60 μL of NaBH4 (0.1 M) was added dropwise to the Ch-Ag solution, and the mixture was stirred for an additional 60 min on ice to form Ch-Ag nanocomposites. Subsequently, the samples were stirred for 30 min at room temperature. Then, the samples were centrifuged for 3 h at 2000 rpm, washed with distilled water, and dried at 105 °C. Finally, chitosan silver nanoparticle composite samples were collected and stored in the lab for further analysis [10].

#### 2.2.3. Characterization of Chitosan-Silver Nanoparticles Composite Materials

In this contribution, the optical, morphological, and structural properties of synthesized chitosan-silver nanoparticle composite materials were characterized using multiple complementary techniques, pertaining to a comparative assessment between the two synthesis methods.

UV-VIS spectroscopy was used to characterize the optical properties and to confirm the formation of silver nanoparticles. Thereafter, the absorption spectra were taken in the range of 190–900 nm using a spectrophotometer (UV-1280, Shimadzu, Kyoto, Japan). The surface plasmon resonance (SPR) band characteristic of silver nanoparticles, which is normally expected around 400–450 nm, was used to verify the successful synthesis and stabilization of silver nanoparticles within the chitosan matrix [1,10]. The intensity and position of SPR peaks were analyzed to contrast nanoparticle size and uniformity differences imparted by the two synthesis methods.

The surface morphology and size distribution of silver nanoparticles contained within the chitosan matrix were investigated using a scanning electron microscope (SEM, S-3400N, Hitachi, Tokyo, Japan). SEM images were recorded and analyzed in a structured image-processing pipeline, following the combination approach of deep-learning-based segmentation and classical morphological analysis developed by Tao et al. [48], to accurately assess the morphology and size distribution of synthesized silver nanoparticles (AgNPs) from SEM micrographs.

A U-Net architecture with a ResNet-50 backbone and Convolutional Block Attention Modules (CBAM) was trained to automatically segment nanoparticles. The training dataset consisted of SEM images together with manually curated masks, where the preprocessing involved resizing, normalization, and data augmentation. After the segmentation step, the predicted masks were post-processed with morphological operations that included the suppression of artifacts, filling holes, and refining particle boundaries.

For scale bar detection, a custom-built automatic algorithm was used to convert pixel-based measurements into physical units (in nanometers). It finds the area of the scale bar in any SEM image, extracts the pixel length, and associates it with the physical value in direct proportion to the nm-per-pixel conversion factor. A particle-wise determination was subsequently carried out through connectivity analysis using regionprops of the scikit-image module to compute the parameters of each particle, including centroid, area, and equivalent diameter. Furthermore, Gaussian smoothing was applied to improve edge definition, and overlay plots for each image were obtained to illustrate the spatial distribution and to validate segmentation accuracy [49].

X-ray diffraction (XRD) analysis was employed using an X-ray diffractometer (XRD, Brucker D8, Bruker AXS GmbH, Karlsruhe, Germany) to study the crystalline structure and phase composition of chitosan-silver nanoparticles produced by the microwave-assisted and one-pot reduction methods. The technique uses the constructive interference of X-rays scattered by periodically arranged atomic planes in crystalline material. Diffraction is thereby produced whenever the incident X-ray beam passes through the sample at a particular angle, and the intensity of rays scattered back from the sample as a function of the incident angle (2theta) produces a diffractogram, or a fingerprint of the crystalline phases of the material [50].

Diffractograms were recorded over the angular range of 10–70° to encompass the angles typically associated with crystalline planes of silver nanoparticles and features of the semi-crystalline chitosan matrix. The range revealed several characteristic Bragg reflections corresponding to metallic silver (notably peaks near 38°, 44°, and 64°, associated with the (111), (200), and (220) planes of face-centered cubic silver and broad features indicating either crystallinity of the chitosan backbone or amorphous regions. The diffractogram patterns were then compared across samples based on their synthesis methods, focusing on differences in peak position, intensity, and width. Changes in peak sharpness and intensity provided insights into the crystalline nature of the samples: sharper and more intense peaks suggest increased crystallinity with well-ordered crystalline domains, while broader peaks indicate more amorphous structures or smaller crystallites.

Raman spectroscopy was employed to analyze the molecular interactions and chemical structures of the nanoparticle composite materials synthesized by microwave-assisted and one-pot reduction methods. The Raman spectra were obtained using a Raman spectrometer (WITec alpha 300R Confocal Raman Microscope, WITec GmbH, Ulm, Germany) equipped with a 532 nm excitation laser within a signal and spectral range of 400 to 4000 cm^−1^, which enabled capturing of the significant vibrational modes associated with the functional groups found in chitosan and their interactions with silver [51]. Therefore, peaks related to C–H, O–H, amide I, and amide II stretching vibrations were monitored for identifying structural modifications attributed to the presence of silver nanoparticles. 

## 3. Results and Discussion

### 3.1. UV-VIS Spectroscopy

**Figure 2:**
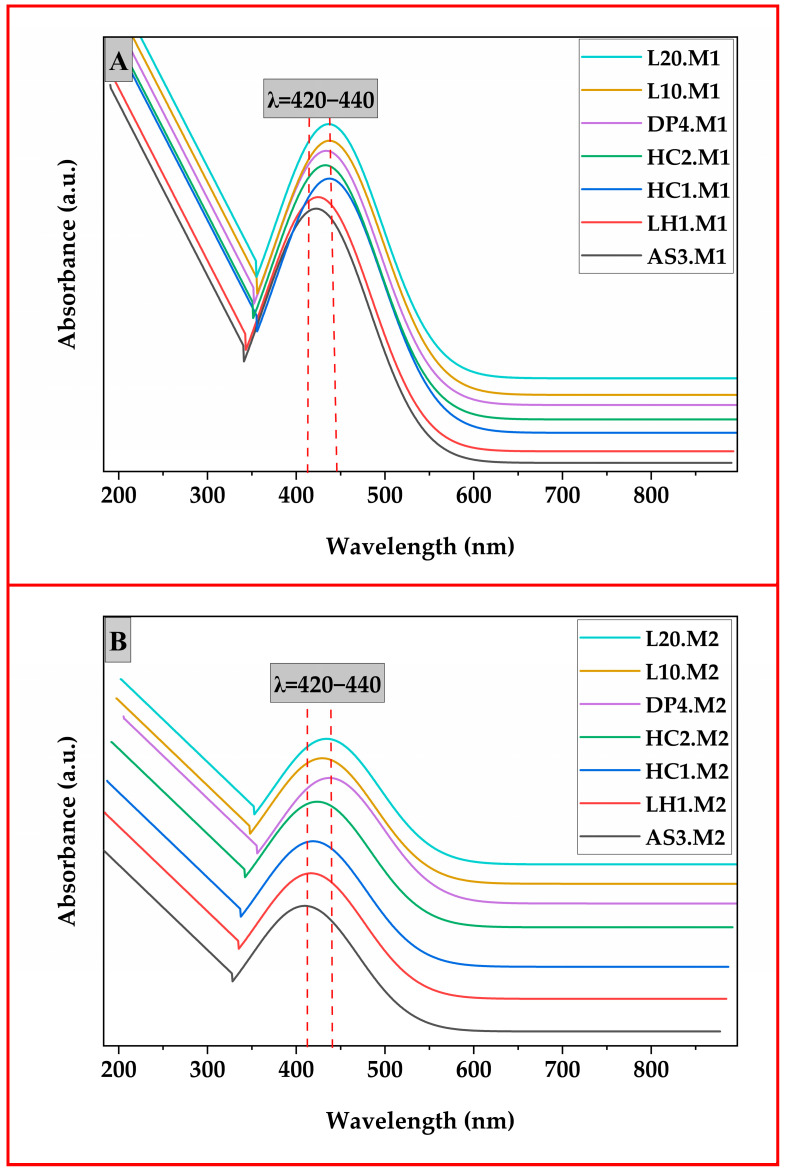
Demonstrates UV-vis spectra of chitosan silver nanoparticle composite materials synthesized via (**A**) microwave-assisted and (**B**) one-pot reduction methods.

As shown in Figure 2, the UV-VIS spectra of the chitosan-based silver nanoparticles (AgNPs) synthesized via microwave-assisted reduction and one-pot reduction methods revealed characteristic surface plasmon resonance (SPR) peaks centered approximately between 400 and 450 nm for all samples, a typical range for spherical AgNPs in the chitosan matrix. In Panel (A), the UV-vis spectra of Method 1 samples depicted that all samples from AS3.M1 to LH20.M1 exhibited sharp and well-defined SPR peaks located between 420 and 440 nm. The presence of narrow, high-intensity SPR bands confirms the successful synthesis of relatively monodisperse and spherical AgNPs with very limited aggregation. The sharpness and consistent position of the SPR peaks suggest an even size distribution. The microwave-assisted route provides a scenario of homogeneous nucleation and growth, upon rapid and uniform heating, leading to a narrow size range of nanoparticles. These observations correlate with the previous research, which stated that microwave heating yields uniformly synthesized and crystalline AgNPs with strong SPR characteristics [52,53]. Conversely, the spectra of the one-pot reduction method samples (AS3.M2 to L20.M2) demonstrated on Panel B showed that the formed nanoparticles exhibited broader, less symmetric SPR peaks, with a slight red shift for some of the samples (L10.M2, L20.M2), attributable to a greater degree of polydispersity and some potential particle aggregation. These Lesser peak intensities and broadening in the spectra from 420 to 450 nm suggest a more heterogeneous size distribution of the nanoparticles and possibly greater aggregation of the nanoparticles, in agreement with previous studies [54,55], which reported that fast reductions via strong reductants such as NaBH4 give rise to polydisperse and less stable particles.

### 3.2. Scanning Electron Microscopy (SEM) and Particle Size Distribution

This study explored the image processing results based on scanning electron microscopy (SEM) micrographs, as shown in Table 2. The SEM images of samples synthesized with microwave assisted method indicated relatively smooth polymer matrices which have clearly distinguishable particles, mostly uniform in shape, signifying its controlled nucleation and growth caused by microwave irradiation; moreover, the Segmentation Output (Predicted Masks) displayed discrete, isolated particles, relatively uniform in size and distribution, which supports the interpretation that particles were well-dispersed and less aggregated. In contrast, the one pot reduction method SEM images showed a rougher, wrinkled, or fibrous surface with hardly identifiable particle boundaries which, taken together, indicate poorly uniform deposition of particles or much possible agglomeration occurring during the chemical reduction in an ice bath, while the Predicted Masks images exhibited denser clusters, less regular in shape, yet more fragmented particles suggesting overlapping broad size distribution and possibly non-uniform nucleation.

On the other hand, particle size distributions of chitosan-silver nanoparticles (Ch-AgNPs) synthesized via method 1, microwave-assisted green synthesis, and method 2, one-pot-reduction method derived from the image processing of SEM micrographs using a deep-learning-enhanced segmentation pipeline, followed by a morphological analysis of the labeled regions (connectivity analysis) in which each segmented particle was individually measured concerning its equivalent diameter.

**Figure 3:**
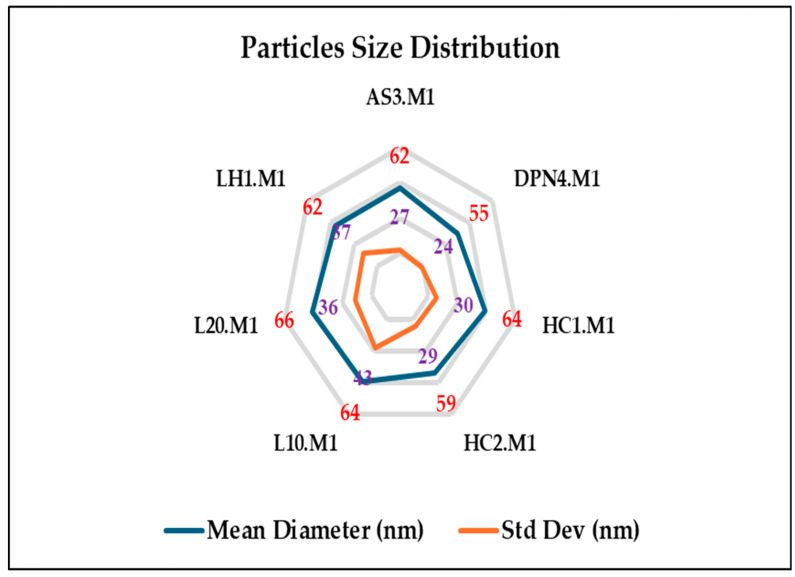
Particle size distribution of chitosan silver nanoparticles synthesized via method 1 (microwave-assisted reduction).

**Figure 4:**
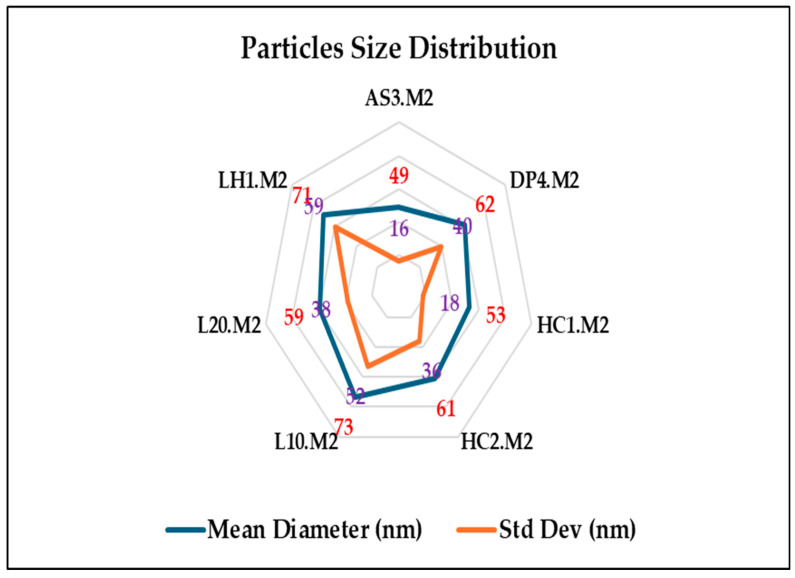
Particle size distribution of chitosan silver nanoparticles synthesized via method 2 (one-pot reduction).

As depicted in Figure 3 and Figure 4. The radar plots visually and comparatively portray the particle size distributions of silver nanoparticles synthesized via method 1, microwave-assisted green synthesis, and method 2, one-pot-reduction method. The radar chart in Figure 3 showed that the microwave-assisted synthesis method produced particles of smaller sizes, implying consistent mean diameters in almost all samples, from ~55 nm (DPN4.M1; the lowest) to ~66 nm (L20.M1; the highest, suggesting a homogeneous synthesis outcome. Standard deviation (Std Dev) values are typically moderate—within ~24 nm to ~43 nm—indicating a moderately polydisperse nanoparticle population. L10.M1 and LH1.M1 samples exhibited slightly wider distributions (Std Dev ~43 and ~37 nm, respectively), which would indicate either aggregation or less controlled nucleation under microwave conditions. The uniformity in particle sizes confirms the effects of microwave irradiation in ensuring rapid, uniform heating, directing controlled crystal growth [25,52].

In contrast, the radar plot in Figure 4 for the one-pot reduction method demonstrated a greater variation in both mean particle diameter values and standard deviations. The mean diameters are more scattered, ranging from ~49 nm (AS3.M2) to ~73 nm (L10.M2), implying inconsistent particle formation within the chitosan matrix, probably because of the rapid and uncontrolled reduction kinetics of NaBH_4_. It was noticed that the standard deviation values of Method 2 are quite significantly higher than those from Method 1 for most samples, reaching ~59 nm (LH1.M2). Such a large deviation signifies a higher level of polydispersity and the possible formation of aggregates or a mixture of small and larger particles. This variance could arise from instantaneously burst nucleation powered by the intense reduction potential of NaBH_4_, followed subsequently by limited control of growth during the synthesis. Furthermore, method 1 exhibited almost unique smaller particle size distributions in different samples, which proves a more suitable condition for creating monodisperse AgNPs, regardless of the chitosan extraction method. Whereas Method 2 seems to be effective in rapidly inducing nucleation, it deteriorates particle size distribution, generating broader ranges with higher discrepancies. This affirms the prior observation using spectroscopy (UV-Vis) and microscopy (SEM) that Method 1 had better control, while in Method 2, there was more heterogeneous nucleation and growth. Such polydispersity in Method 2 could affect physicochemical stability and functionalities of nanoparticles, which is very critical for antimicrobial applications where uniformity in the size of particles leads to enhancement in surface reactivity and better biocompatibility [1].

### 3.3. Raman Spectroscopy

**Figure 5:**
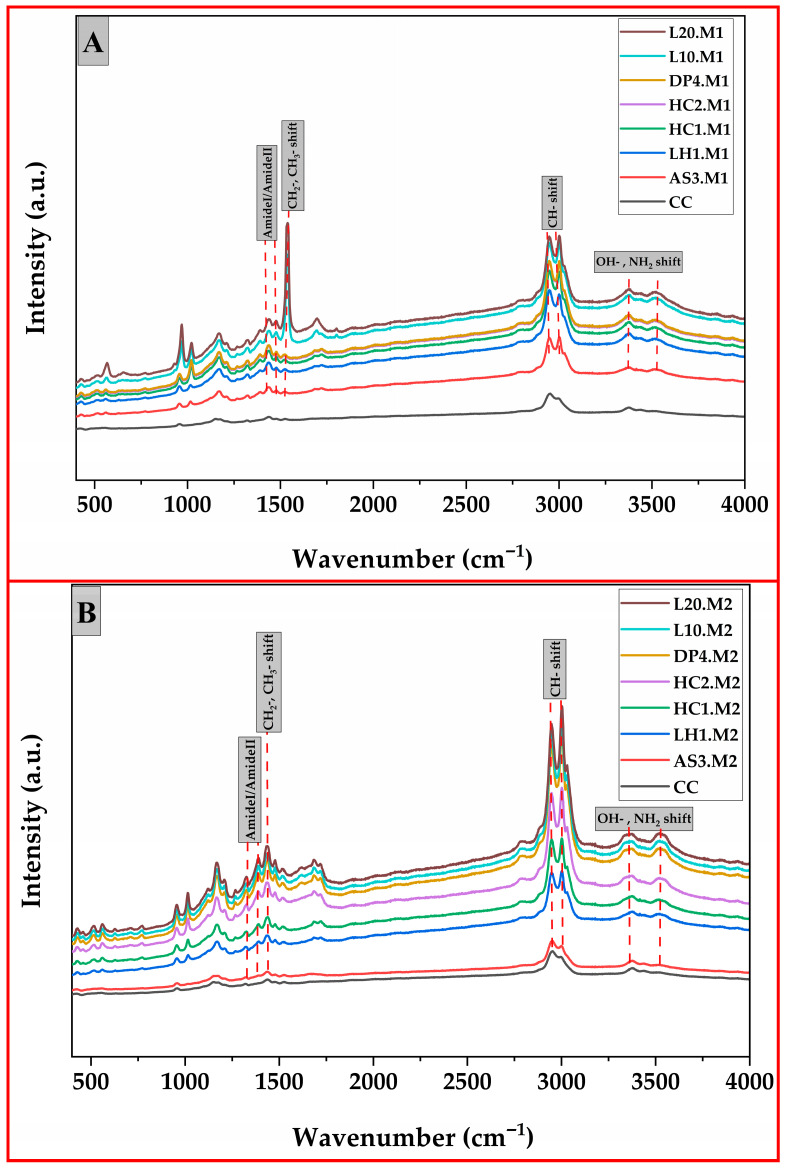
Raman shifts of chitosan silver nanoparticles synthesized (**A**) via method 1 (microwave-assisted reduction) and (**B**) method 2 (one-pot reduction method).

The molecular structure, crystallinity, and surface interactions of the two distinct green synthesis methods for producing silver nanoparticles (AgNPs), microwave-assisted reduction and one-pot reduction methods, were studied using Raman spectroscopy, as shown in Figure 5. The solid-state structures of the samples were compared against a control chitosan (CC) sample, examining the differences imparted by each synthesis method. Both methods of chitosan silver nanoparticle composite materials exhibited Raman spectra with a broad band between 2900 and 3000 cm^−1^, corresponding to C–H and O–H stretching vibrations [56]. In fact, this band is significantly stronger in AgNP-loaded samples compared to control chitosan (CC) because of increased scattering resonance from the embedded AgNPs, a phenomenon generally attributed to the MERS effect in silver–polymer systems.

The Raman spectra of microwave-assisted samples (Figure 5A) showed distinct and very intense vibrational bands in the region 500–1800 cm^−1^ as well as the presence of an obvious peak of ~1375 cm^−1^ and ~1580 cm^−1^, which corresponded to the CH_3_/CH_2_ deformation and amide I/II vibrations of the polymer backbone of chitosan. The sharpness and intensity of these bands suggest a high degree of structural order and effective capping of AgNPs by chitosan molecules. Moreover, the pronounced band around ~2900–3000 cm^−1^, attributed to C–H symmetric and asymmetric stretching, reflects well-organized surface chemistry and uniform interactions between AgNPs and chitosan ligands. These spectral characteristics reflect a high crystallinity and an even formation of nanoparticles and would agree with the SEM analysis of particle size distribution and sharp surface plasmon resonance (SPR) bands in UV-Vis spectra. These vibrational characters are comparable with the biopolymer-capped structurally uniform silver nanoparticles reported in previous studies [56,57].

In contrast, Raman bands of the one-pot reduction method samples (Figure 5B), were broader and less intense, especially in the 500–1800 cm^−1^ range, with a resolution that was significantly lower than that achieved from method 1. In this region, the peaks were apparently broader and less resolved, suggesting rather poor crystallinity and high disorder in the structural solid-state. Broadening of such features can be related to the fact that sodium borohydride yields enhanced and rapid reaction kinetics for the formation of heterogeneous population sizes of nanoparticles, together with less-defined surface coordination [10]. Also, this might explain why the one-pot reduction method lacked precisely defined Ag-O or Ag-N vibrational modes, as the very rapid reduction limited complexation time, favoring particle nucleation over chitosan coordination. Thus, studies support the finding that rapid reduction by NaBH_4_ normally leads to amorphous, polydisperse AgNPs, particularly with non-equilibrium reducing conditions such as lowered temperature and a strong reducing environment [58].

Comparative Raman analysis revealed that the microwave-assisted method yielded more crystalline silver nanoparticles with more homogenous capping than the one-pot reduction method. These findings are supported by narrower UV-Vis spectra in Method 1 and were corroborated by lower standard deviations and tighter size ranges from SEM-based particle size distributions, confirming that microwave-assisted syntheses were indeed better in this respect.

### 3.4. X-Ray Diffraction (XRD) Pattern

**Figure 6:**
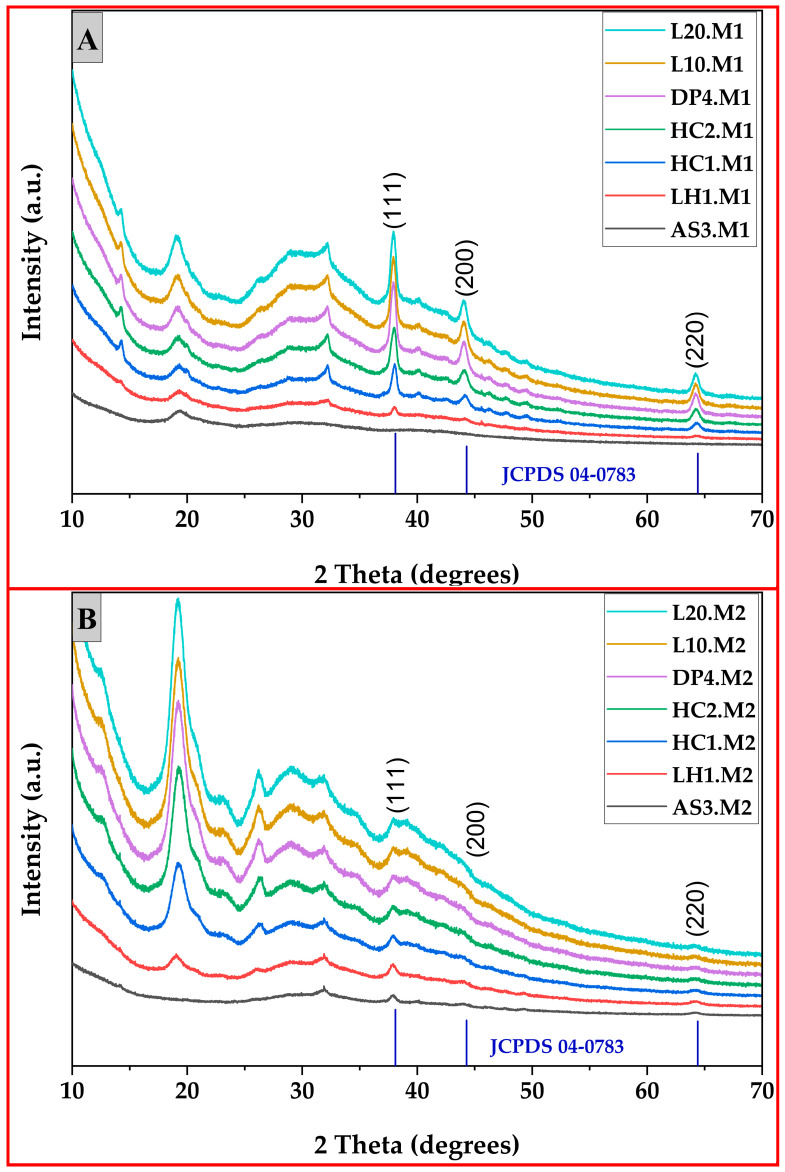
XRD pattern of synthesized chitosan silver nanoparticles (**A**) via method 1 (microwave-assisted reduction) and (**B**) method 2 (one-pot reduction method).

The X-ray diffraction (XRD) patterns of the synthesized chitosan-silver nanoparticle composites produced via method 1 (microwave-assisted synthesis) and method 2 (one-pot reduction) are shown in Figure 6. In both cases of the microwave-assisted and one-pot reduction routes, the broad peak at 2θ ≈ 20° corresponds to the amorphous chitosan backbone, a common feature in similar biopolymer-metal nanocomposites. The samples synthesized via microwave-assisted reduction method (M1) exhibited well-defined diffraction peaks at approximately 2θ = 38°, 44°, and 64°, which correspond to the (111), (200), and (220) lattice planes of the face-centered cubic (FCC) structure of silver, respectively, as shown in Figure 6A. These peaks are in agreement with the Joint Committee on Powder Diffraction Standards (JCPDS) card no. 04-0783 for silver. These peaks are all very sharp and prominent, which is indicative of highly crystalline AgNPs with a main orientation in the (111) direction, usually associated with thermodynamically favorable nanoparticle growth under controlled energy input. This was especially evident in L10.M1 and L20.M1, which exhibited the sharpest and most intense (111) reflections, indicating larger and well-ordered crystallites.

Sample-wise, DP4.M1, HC1.M1, and HC2.M1 also showed well-defined FCC peaks but with lower intensity as compared to that of L20.M1, perhaps because of the smaller size of crystallites or moderate surface disorder. Sample AS3.M1 and LH1.M1 exhibited broadened peaks, indicating partially decreased crystallinity or slight size polydispersity, but generally retained their FCC signatures. As for all the M1 samples, they benefited from the fast and uniform volumetric heating effect of microwave irradiation that encourages homogeneous nucleation and, as a result, promotes crystallite growth.

On the contrary, the samples of M2 (Figure 6B) exhibited broad and less intense diffraction peaks, especially at about 38° and 44°, which correspond to the diffraction planes of (111) and (200), respectively. The broadening and the suppression in intensity of these peaks are indicative of a smaller crystallite size within the samples, as well as a higher amount of strain and amorphous material, which have been attributed to residual biopolymer (chitosan) or rapid nucleation conditions. Specifically, there was a significant loss of peak intensity and the mean widths of the bases for AS3.M2 and LH1.M2, indicating they are poorly crystalline with possible spinel structural heterogeneity.

Other samples like L10.M2 and DP4.M2 represented distinguishable FCC peaks, though their reduced sharpness compared to their M1 counterparts points toward smaller or more polydisperse nanostructures. This is consistent with the higher standard deviation values observed in the image-based particle size analysis. Notably, the broad hump between 20° and 30° in several M2 samples may originate from amorphous chitosan residues or organic reaction intermediates, which remained unremoved due to the absence of post-synthetic thermal treatment.

Such structural differences tend to confirm that microwave-assisted synthesis produces a more crystalline and well-structured nanoparticle because of controlled thermal kinetics, whereas cold one-pot reduction causes rapid nucleation but prevents materials from growing as crystals over time, leading to more disordered nanostructures. Supported by parallel data from UV-Vis absorbance (which showed narrower surface plasmon resonance bands in M1) and SEM-based particle analysis, the M1 samples consistently exhibited smaller standard deviations and more uniform shapes.

However, some previous research [58] reported that the induction and long-term exposure to X-ray irradiation can cause the formation or structural changes in reactive metallic nanostructures within the polymeric matrix under certain conditions. In our current study, we considered such effects to minimize the risk of growth or morphological changes resulting from X-ray exposure by stabilizing the measurement conditions and shortening the exposure time in order to confirm the crystalline nature of the synthesized chitosan silver nanoparticles.

## 4. Conclusions and Future Research Directions

This comparative study systematically investigated green synthesis strategies for silver nanoparticles (AgNPs) utilizing chitosan as a stabilizing agent: Method 1 comprised a microwave-assisted reduction using ascorbic acid, while Method 2 followed a one-pot ice-bath reduction using sodium borohydride. Characterizations of the obtained AgNPs in terms of structure, morphology, and optical property were carried out using UV-Visible spectroscopy, scanning electron microscopy (SEM), automated image-based particle size analysis, Raman spectroscopy, and X-ray diffraction (XRD).

UV-Vis analysis showed that Method 1 produced more uniform and well-dispersed nanoparticles with narrower surface plasmon resonance peaks around 430 nm. Whereas Method 2 caused broader, red-shifted peaks indicative of greater particle size variation and agglomeration. These corresponding optical feature results were verified by SEM imaging along with image-based particle size assessments; Method 1 confirmed smaller and more monodispersed particles. Moreover, Raman Spectroscopy and XRD were of much importance in building the material view of the structure morphology. For instance, Method 1 samples showed sharper Raman bands with well-defined XRD peaks of higher intensity, thus indicating the nanoparticles to be more crystalline and stabilized better by the chitosan, while the Raman spectra for Method 2 were much broader, with less intense diffraction data, features that therefore exhibited more disorder and a polydisperse nanoparticle population. Thus, it establishes superiority in terms of uniformity and crystallinity of nanoparticles synthesized using the microwave-assisted method.

Based on the findings of this comparative study, the application of chitosan-based silver nanoparticles synthesized via a microwave-assisted approach as antimicrobial agents for sustainable crop protection can be a more promising potential alternative in the agronomy sector, rather than traditional chemical pesticides, minimizing the increased threats posed by plant pathogens. These environmentally benign and structurally uniform chitosan AgNPs, fabricated through a microwave-assisted method, would become ideal candidates, since silver nanoparticles have emerged as a potent alternative due to their broad-spectrum antimicrobial properties. More research is needed to evaluate the antimicrobial efficacy of these nanoparticles in vivo against common phytopathogens, ensuring their biocompatibility with plant tissues, effective dosage, and delivery systems, environmental safety, and crop yields. Equally, the exploration of synergistic formulation with other biostimulants or micronutrients could open a horizon towards smart nanocomposite fertilizers and agro-defense materials.

## Figures and Tables

**Table 1:** Properties of chitosan samples isolated from shrimp shells.

Sample	Moisture Content (%)	Ash Content (%)	Degree of Deacetylation (DD%)
AS3	0.34	3.5	99.35
LH1	0.56	2.6	99.40
HC1	1.32	0.90	91.1
HC2	1.35	0.76	91.3
DP4	0.19	0.92	99.24
L10	2	1.06	99.42
L20	2.74	0.70	99.38

**Table 2:** SEM and particle segmentation comparison between nanoparticles synthesized by microwave-assisted (M1) and one-pot reduction (M2) methods.

Sample ID	SEM.M1	Particle SegmentationM1	SEM.M2	Particle SegmentationM2
AS3	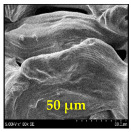	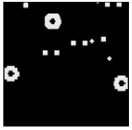	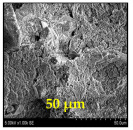	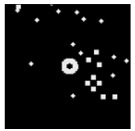
DP4	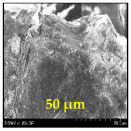	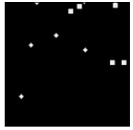	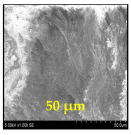	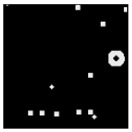
HC1	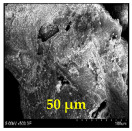	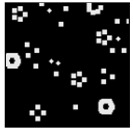	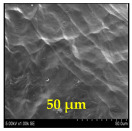	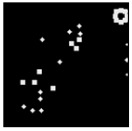
HC2	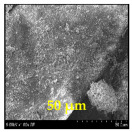	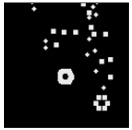	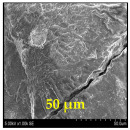	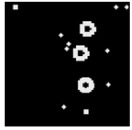
L10	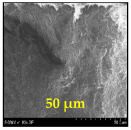	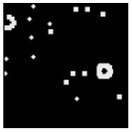	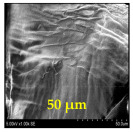	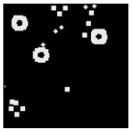
L20	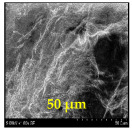	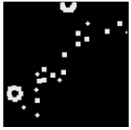	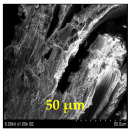	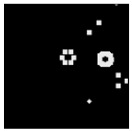
LH1	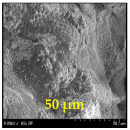	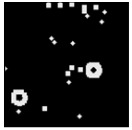	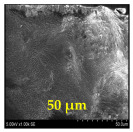	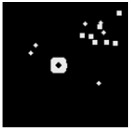

## Data Availability

The original contributions presented in this study are included in the article. Further inquiries can be directed to the corresponding authors.
